# Use of D-glucose–fenpiclonil conjugate as a potent and specific inhibitor of sucrose carriers

**DOI:** 10.1093/jxb/erx354

**Published:** 2017-10-27

**Authors:** Hanxiang Wu, Sophie Marhadour, Zhi-Wei Lei, Émilie Dugaro, Cécile Gaillard, Benoit Porcheron, Cécile Marivingt-Mounir, Rémi Lemoine, Jean-François Chollet, Jean-Louis Bonnemain

**Affiliations:** 1 Laboratoire EBI (Écologie et Biologie des Interactions), UMR CNRS 7267, Équipe SEVE (Sucres, Échanges Végétaux, Environnement), Université de Poitiers, 3 rue Jacques Fort, Poitiers cedex, France; 2 IC2MP (Institut de Chimie des Milieux et des Matériaux de Poitiers), UMR CNRS 7285, Université de Poitiers, 4 rue Michel Brunet, TSA, Poitiers cedex, France; 3 Guizhou Tea Reasearch Institute, Guizhou Academy of Agricultural Science, Guiyang, Guizhou, China

**Keywords:** Apoplasmic loaders, Arabidopsis AtSUC2, D-glucose–fenpiclonil conjugate, PCMBS, phloem loading, *Ricinus* seedlings, specific inhibition of sucrose carriers, sucrose uptake from endosperm, *Vicia faba*

## Abstract

Until now, specific inhibitors of sucrose carriers were not available. This led us to study the properties of the recently synthesized D-glucose–fenpiclonil conjugate (D-GFC). This large amphiphilic glucoside exhibited an extremely low phloem systemicity in contrast to L-amino acid–fenpiclonil conjugates. Using *Ricinus* seedlings, the effect of D-GFC on 0.5 mM [^14^C]sucrose (Suc), 3-*O*-[^3^H]methylglucose, and [^3^H]glutamine uptake by cotyledon tissues was compared with that of *p*-chloromercuribenzenesulfonic acid (PCMBS). D-GFC dramatically inhibited H^+^–Suc symport at the same concentrations as PCMBS (0.5 and 1 mM), but in contrast to the thiol reagent, it did not affect 3-*O*-methylglucose and glutamine transport, nor the acidification of the incubation medium by cotyledon tissues. Similarly, 0.5 mM D-GFC inhibited active Suc uptake by *Vicia faba* leaf tissues and by *Saccharomyces cerevisiae* cells transformed with *AtSUC2*, a gene involved in Suc phloem loading in Arabidopsis, by approximately 80%. The data indicated that D-GFC was a potent inhibitor of Suc uptake from the endosperm and of Suc phloem loading. It is the first chemical known to exhibit such specificity, at least in *Ricinus*, and this property permitted the quantification of the two routes involved in phloem loading of endogenous sugars after endosperm removal.

## Teaser

A D-glucose conjugate from the phenylpyrrole family potently inhibits active uptake and phloem loading of sucrose in plants from different families. Its high specificity in *Ricinus* allows new investigations in phloem biology.

## Introduction

The non-permeant or poorly permeant sulfhydryl reagent *p*-chloromercuribenzenesulfonic acid (PCMBS) has been successfully used in phloem biology, first to demonstrate that sucrose accumulates in the phloem symplasm from the vein apoplasm through Suc carriers as in *Beta vulgaris* and *Vicia faba* (Giaquinta, 1976; Delrot *et al.*, 1980; Giaquinta, 1983), and then to identify apoplasmic and symplasmic loaders (van Bel *et al.*, 1992; Turgeon and Medville, 2004; Turgeon and Ayre, 2005). PCMBS dramatically inhibits the activity of Suc carriers but because it reacts with cysteine residues of many other plasma membrane (PM) intrinsic proteins, it also affects the transport of other solutes, as demonstrated in *Ricinus communis* (Williams *et al.*, 1996; Rocher *et al.*, 2009; Tamas and Davies, 2016). The effect of PCMBS on the PM H^+^-ATPase varies with tissues. For instance, it does not significantly affect the proton pumping activity nor the transmembrane potential difference in mature broad bean leaf tissues (Delrot *et al.*, 1980; Bourquin *et al.*, 1990), but it inhibits proton pumping by microsomal vesicles and acidification of the incubation medium by intact cotyledons of *Ricinus communis* seedlings (Williams and Hall, 1987; Williams *et al.*, 1990).

The *Ricinus* seedling has been successfully used as a model plant to study the composition of the phloem sap (Kriedemann and Beevers, 1967; Schobert and Komor, 1989; Vreugdenhil and Koot-Gronsveld, 1989; Gerendas and Schurr, 1999; Kallarackal *et al.*, 2012), the mechanisms of nutrient uptake by cotyledon tissues and phloem loading (Komor *et al.*, 1977; Komor *et al.*, 1980; Robinson and Beevers, 1981*b*; Marvier *et al.*, 1998; Williams *et al.*, 1996), as well as the long-distance transport of sugars (Kallarackal *et al.*, 1989; Metzler *et al.*, 1994; Verscht *et al.*, 1998; Kallarackal *et al.*, 2012). As in most plant species (Liu *et al.*, 2012), sucrose (Suc) is the major sugar of the *Ricinus* phloem sap, with concentrations of approximately 300 mM in intact seedlings (Kallarackal *et al.*, 1989; Verscht *et al.*, 1998). Glucose and fructose have much lower concentrations (approximately 2 and 0.6 mM, respectively) (Kallarackal and Komor, 1989). However, the phloem of *Ricinus* seedlings exhibits the peculiarity of loading exogenous glucose. When cotyledons are dipped in solutions containing from 25 to 200 mM glucose, the same concentrations as in the incubation solutions are found in the phloem sap after 2 h of incubation (Kallarackal and Komor, 1989).

**Fig. 1.**
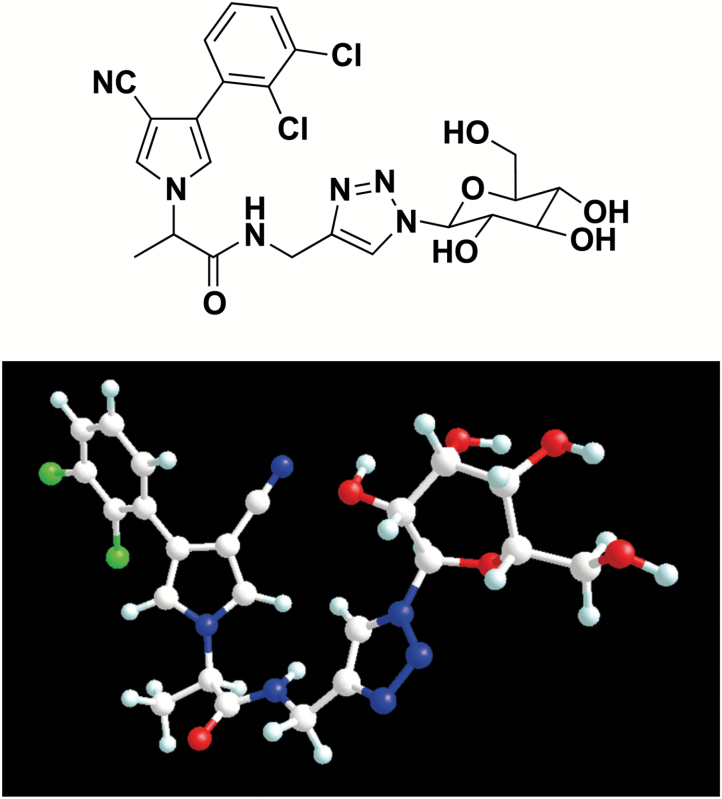
Two- and three-dimensional structure of D-GFC obtained using Chem3D Pro, energy minimization with the MM2 method. Atoms are denoted by spheres in the following colours: carbon in pale grey, hydrogen in light blue, chlorine in green, oxygen in red, and nitrogen in blue. For this compound, *M*_r_=551.38 and *K*_ow_=0.71 (computed with ACD/Labs Percepta 2015 release).

The *Ricinus* seedling has also been used to study the phloem mobility of xenobiotic conjugates, i.e. compounds that associate an agrochemical and an α-amino acid (Dufaud *et al.*, 1994; Chollet *et al.*, 1997; Delétage-Grandon *et al.*, 2001; Wu *et al.*, 2016; Xie *et al.*, 2016) or a monosaccharide (Yang *et al.*, 2011; Wu *et al.*, 2012; Yuan *et al.*, 2013) in their structure. This vectorization strategy has been developed to evaluate the ability of PM carriers to translocate large and halogenated xenobiotics (Delétage-Grandon *et al.*, 2001; Yang *et al.*, 2011; Wu *et al.*, 2012; Yuan *et al.*, 2013). We have recently synthesized two types of conjugate of fenpiclonil, a contact fungicide from the phenylpyrrole family used as a model molecule, namely, a D-glucose conjugate and an L- and a D-glutamic acid conjugate (Wu *et al.*, 2016). These compounds, which violate both Lipinski’s (Lipinski *et al.*, 2012) and Veber’s (Veber *et al.*, 2002) rules, were predicted to have very low membrane permeability. Nevertheless, they were found in the phloem sap. Systemicity tests using the *Ricinus* model indicated that these large chlorinated conjugates exhibited dramatic differences in their ability to move in the phloem. When cotyledons were dipped in an incubation solution buffered at pH 5.0, the concentrations of the D-glucose conjugate and the D-glutamic acid conjugate in the phloem sap were 20 and 5 times lower than that of the L-glutamic acid conjugate, respectively. The phloem systemicity of the fenpiclonil glucoside was even 30–45 times lower than that of the most recent L-amino acid–fenpiclonil conjugates synthesized (Marhadour *et al.*, 2017). Depending on their structure, natural glucosides exhibit different abilities to move in the phloem. For instance, glucosinolates are translocated long distance within the plant (Chen *et al.*, 2001; Turgeon and Wolf, 2009), and two members of the nitrate/peptide transporter family (GTR1 and GTR2) are involved in the phloem loading of these defence compounds (Nour-Eldin *et al.*, 2012). Small and hydrophilic glucosides such as salicin (*M*_r_=286.28; log *P*=−1.48) and arbutin (*M*_r_=272.25; log *P*=−1.14) are translocated by AtSUC2 expressed in *Xenopus laevis* oocytes (Chandran *et al.*, 2003). By contrast, the presence of phlorizin in the phloem sap has not been reported until now. This glucoside of phloretin is a non-transported competitive inhibitor of Na^+^–glucose cotransporters in animal cells (Toggenburger *et al.*, 1982; Hummel *et al.*, 2011). In plants, phlorizin is recognized by hexose and Suc carrier systems but more efficiently inhibits Suc phloem loading than hexose uptake in broad bean leaves (Lemoine and Delrot, 1987). Due to the glycosyl hydroxyls (Hitz *et al.*, 1986; Delrot *et al.*, 1991), D-GFC may be recognized by Suc carriers but the size (*M*_r_=551.38) and the amphiphilic structure (Fig. 1) of the compound are completely inappropriate for translocation considering the molecular structural requirements suitable for Suc carrier activity (Hitz *et al.*, 1986; Delrot *et al.*, 1991; Chandran *et al.*, 2003). Therefore, we hypothesized that D-GFC would affect sugar translocation systems. We use *Ricinus* as our model to test this hypothesis because it can load in the phloem not only endogenous Suc but also exogenous hexoses as mentioned above.

The purpose of this work was initially to compare the effect of D-GFC and PCMBS on Suc, 3-*O*-methylglucose (3-*O*-MeG) and glutamine (Gln) uptake and phloem transport in *Ricinus* seedlings. The results allowed a quantitative study of the contribution of the two pathways involved in phloem loading after endosperm removal and led us to extend the investigation to other biological models.

## Materials and methods

### Plant material

Castor bean seeds (*Ricinus communis* L. cv Sanguineus) were grown as previously described (Delétage-Grandon *et al.*, 2001). After 6 days of growth in vermiculite, seedlings of average size were selected for the experiments.

Broad bean (*Vicia faba* cv Aguadulce) plants were grown on vermiculite and watered daily with a nutrient solution as already described (Lemoine *et al.*, 1984). The experiments were performed on plants possessing five mature bifoliate leaves.

*Saccharomyces cerevisiae* strain RS453 cells were grown and transformed as described in Henry *et al.* (2011).

### Chemicals

We have previously described the detailed synthesis of the D-glucose–fenpiclonil conjugate (D-GFC; Fig. 1) (Wu *et al.*, 2016). This conjugate was obtained using click chemistry, by coupling fenpiclonil, a fungicide from the phenylpyrrole family, to D-glucose via a spacer group including a 1,2,3-triazole ring.

PCMBS was purchased from Toronto Research Chemicals Inc., 3-*O*-[^3^H]MeG (60 Ci mmol^−1^) was purchased from Isobio and [^3^H]Gln (50.1 Ci mmol^−1^) and [U-^14^C]Suc (435 mCi mmol^−1^) were purchased from PerkinElmer SAS.

### Uptake and exudation experiments with *Ricinus* seedlings

The cotyledons were preincubated in the standard solution buffed at pH 5.0 (Rocher *et al.*, 2006). Then cotyledons were incubated in the same solution without or with D-GFC at 0.5 mM concentration for 30 min (10 ml per plant). Then, a mixture of unlabelled Suc and [^14^C]Suc, unlabelled 3-*O*-MeG and 3-*O*-[^3^H]MeG or unlabelled Gln and [^3^H]Gln was supplied to the solution to obtain a 0.5 mM final concentration and a specific activity of 0.04 mCi mmol^−1^, 0.30 mCi mmol^−1^ or 0.30 mCi mmol^−1^, respectively. Thirty minutes later, the hypocotyl was cut in the hook region for phloem exudation of the exogenous solutes. The phloem sap was collected from the upper part of the hypocotyl for 5 h and was stored at −20 °C until analysis. Phloem sap was added to 4 ml scintillation cocktail (EcoLite, ICN Biomedicals). Radioactivity was measured with a liquid scintillation analyser (Tri-Carb 2910TR, PerkinElmer).

At the end of the exudation period, the cotyledons were rinsed with the buffer solution (3 × 2 min), wiped off with filter paper and weighed. Then, the cotyledons were digested overnight at 60 °C in a mixture of perchloric acid (65%; 0.56 ml), hydrogen peroxide (33%; 0 27 ml), and Triton X-100 (1 g l^−1^; 0.17 ml). Radioactivity measurements were conducted on each seedling separately.

### Analysis of endogenous sucrose, glucose, and fructose in *Ricinus* phloem sap

The *Ricinus* cotyledons were incubated in buffer solution (from pH 5.0 to 8.0) containing 0.25 mM MgCl_2_ and 0.5 mM CaCl_2_. The buffer used was 20 mM MES (pH 5.0 and 6.0) or 20 mM HEPES (pH 7.0 and 8.0) (Rocher *et al.*, 2006). The phloem sap was collected from the upper part of the *Ricinus* seedlings according to the methods already described (Kallarackal *et al.*, 1989). After removing the endosperm, the cotyledons of intact seedlings were preincubated for 30 min in the buffer solution. Then the cotyledons were incubated in the same buffer solution with or without 0.5 mM D-GFC. Thirty minutes later, the hypocotyl was cut in the hook region and the collected phloem sap was stored at −20 °C until analysis.

The endogenous sugars glucose, fructose, and Suc in the phloem sap were determined enzymatically using previously described methods (Orlich and Komor, 1992). A Suc/D-fructose/D-glucose assay kit (K-SUFRG; Megazyme, Ltd, Bray, Ireland) was used following the manufacturer’s instructions. To measure the Suc concentration, the phloem sap was diluted 100-fold with pure water. All measurements were performed by using 1/4 of the amounts of the reagents recommended by the manufacturer.

### pH transients in the incubation solution of *Ricinus* seedlings

The measurement of pH transients in the medium using *Ricinus* cotyledons was similar to that described previously (Komor *et al.*, 1977; Hutchings, 1978; Robinson and Beevers, 1981*b*). The cotyledon still attached to the seedling was incubated in a solution (10 ml) containing 0.25 mM MgCl_2_ and 0.5 mM CaCl_2_. The solution was stirred continuously and the pH of the solution was monitored every 30 s with a microelectrode and a pH meter (S220 SevenCompact pH/Ion Meter, Mettler Toledo). The pH of the solution bathing the cotyledons decreased to pH 4.6–4.9 after 30 min then stabilized between 4.8 and 5.0. Small aliquots of concentrated solutions of D-GFC or PCMBS (1 mM final concentration) were added at 30 min and small aliquots of concentrated solutions of Suc (20 mM final concentration) were added at 60 min. The pH was monitored continuously over 3 h.

### Uptake experiments with leaf discs of broad bean

The leaf discs of broad bean were prepared as previously described (Lemoine and Delrot, 1987). After stripping off the lower epidermis, leaf discs (1.13 cm^2^) were preincubated for 30 min in a buffer solution containing 0.25 mM MgCl_2_, 0.5 mM CaCl_2_, 250 mM mannitol, and 20 mM MES (pH 5.0). The discs were then incubated in the same buffer solution with or without 0.5 mM inhibitor (D-GFC or PCMBS) in the presence of a mixture of unlabelled and ^14^C-labelled Suc (final concentration: 0.5 mM; specific activity: 0.20 mCi mmol^−1^) or a mixture of unlabelled and ^3^H-labelled 3-*O*-MeG (final concentration: 0.5 mM; specific activity: 0.30 mCi mmol^−1^) for 30 min (20 ml per 15 discs). At the end of incubation the discs were rinsed in a solution similar to the preincubation medium (3 × 2 min). Each disc was then digested overnight at 60 °C in a mixture of perchloric acid (65%; 112 µl), hydrogen peroxide (33%; 54 µl), and Triton X-100 (1 g l^−1^; 34 µl). Radioactivity was measured by the liquid scintillation analyser mentioned above. The measurements were made on each disc separately.

### Uptake experiments in yeast

The *AtSUC2* coding region in the plasmid pDONR207 coding region was a generous gift from Dr F. Vilaine (Insitut Jean Pierre Bourgin, Versailles, France). The coding region was cloned by recombination into plasmid pDR-R1-R2-HIS3 (Cagnac *et al.*, 2007) derived from pDR 192. The plasmid containing *AtSUC2* and the empty plasmid were inserted into *Saccharomyces cerevisiae* RS453 and Suc uptake experiments were run as described in Henry *et al.* (2011). Briefly, yeast cells were grown to early logarithmic phase in YNB medium supplemented with 2% glucose. Cells were washed and resuspended with 50 mM MES buffer (pH 4.5) to reach a final OD_600nm_ value of 0.5. Aliquots (100 µl) of cell suspension were added to 100 µl of a solution containing 50 mM MES (pH 4.5) and a mixture of unlabelled and ^14^C-labelled Suc (concentration: 1 mM; specific activity: 0.50 mCi mmol^−1^) at 28 °C for 5 min. The final sucrose concentration in the medium was therefore 0.5 mM.

The reactions were stopped by adding 8 ml of cold water and immediate filtration on glass microfibre filters (25 mm, Fisher Bioblock, Illkirch, France). This step was repeated once. Radioactivity incorporated into cells collected on filters was evaluated using a liquid scintillation counter.

## Results and discussion

### Effect of the D-glucose–fenpiclonil conjugate on the uptake and phloem transport of exogenous Suc, 3-*O*-MeG, and Gln in *Ricinus* seedlings in comparison with PCMBS

The two nutrients and the sugar analogue were selected because of their high concentrations in the phloem sap and/or their poor metabolism. Suc is slowly metabolized in *Ricinus* cotyledon tissues (Kriedemann and Beevers, 1967; Komor *et al.*, 1977) and represents 98–99% of the total sugar in the phloem sap (Kallarackal and Komor, 1989). The mobile analogue 3-*O*-MeG is slowly phosphorylated in plant tissues (Cortès *et al.*, 2003). Chromatography analyses suggest the absence of metabolic transformation of 3-*O*-MeG in the *Ricinus* phloem exudate under short-time experiments (2 h) (Kallarackal and Komor, 1989). Gln is the major amino acid found in the *Ricinus* phloem sap, comprising 30–40% of the total amino acids (Robinson and Beevers, 1981*a*; Schobert and Komor, 1989) and dramatically accumulates in the phloem sap from the apoplast between the endosperm and the cotyledons (endogenous Gln) or from an incubation solution (exogenous Gln) (Schobert and Komor, 1989). The cotyledon tissues acidified the non-buffered solution to pH values of 4.8–5.0 as mentioned below. Therefore the experiments were conducted using incubation solutions buffered at pH 5.0.

**Fig. 2.**
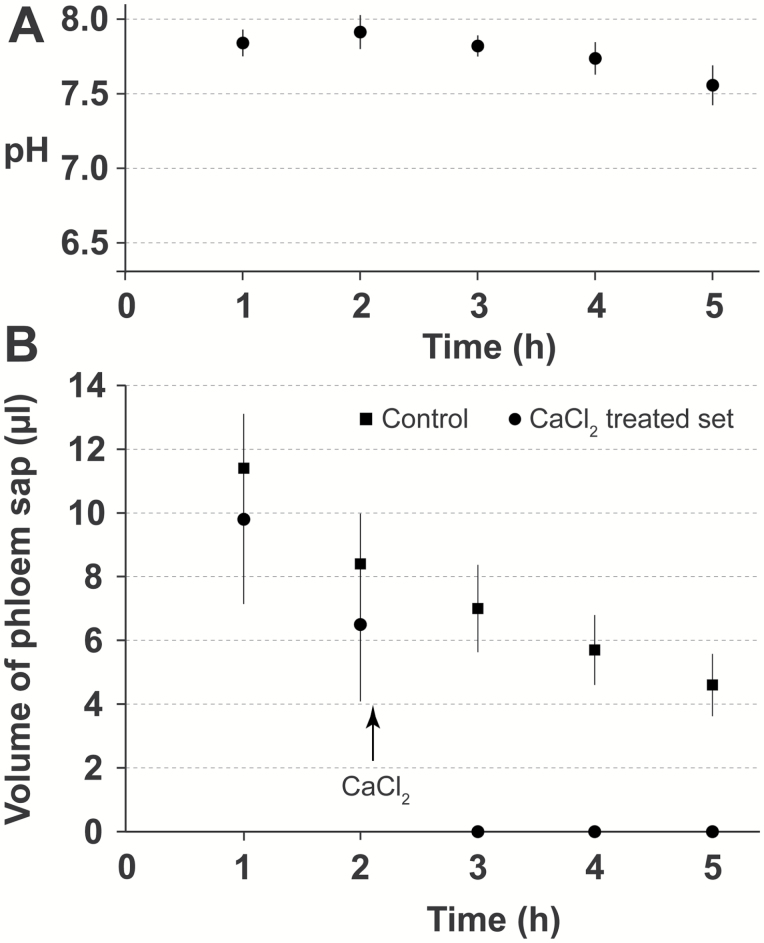
Phloem sap exudation from *Ricinus* seedlings. (A) pH time course variation of the *Ricinus* phloem sap. After preincubation for 60 min in a standard medium (pH 5.0), the hypocotyls were severed, and then the phloem sap was collected each hour from the upper part for pH measurements. Each point is the mean±95% CI of three sets of five plants. (B) Effect of 1 M CaCl_2_ on sap exudation in the presence of D-GFC. After preincubation for 30 min in a standard medium (pH 5.0), the cotyledons were dipped in the same solution containing 0.5 mM D-GFC for 30 min. Then the hypocotyls were severed and the phloem sap was collected each hour from the upper part for volume measurements. In the treated sets, 1 M CaCl_2_ solution was applied to the hypocotyl section just after the second sap collection (arrowhead). Each point is the mean of 10 plants±95% CI.

Contrary to species of the Cucurbitaceae (Zhang *et al.*, 2012; Zimmermann *et al.*, 2013), after *Ricinus* hypocotyls were cut under our experimental conditions, an immediate and strong efflux of a mixture of phloem and xylem sap did not occur from the apical side of the cut. The concentration of Suc, which was approximately 300 mM in intact seedlings, decreased to approximately 100 mM 2 h after endosperm removal (Kallarackal and Komor, 1989) and to approximately 160 mM in our plant material. The concentrations of glucose and fructose were very low, approximately 2 and 0.7 mM, respectively. The concentrations of these three sugars remained stable for at least 5 h as detailed below. The pH of the sap remained almost unchanged (7.9–7.6) (Fig. 2A). After addition of 1 M CaCl_2_, which induced a biphasic occlusion of sieve tubes (Furch *et al.*, 2010), no fluid in measurable quantities was released from the apical cut until the end of the experiment. The result was the same in the presence of D-GFC (Fig. 2B). All the data suggest that the phloem sap is very poorly contaminated by the apoplasmic fluid despite long incubation times in solution and indicate that phloem sap could be collected for at least 5 h after the first hour of preincubation, i.e. 6 h after endosperm removal.

**Fig. 3.**
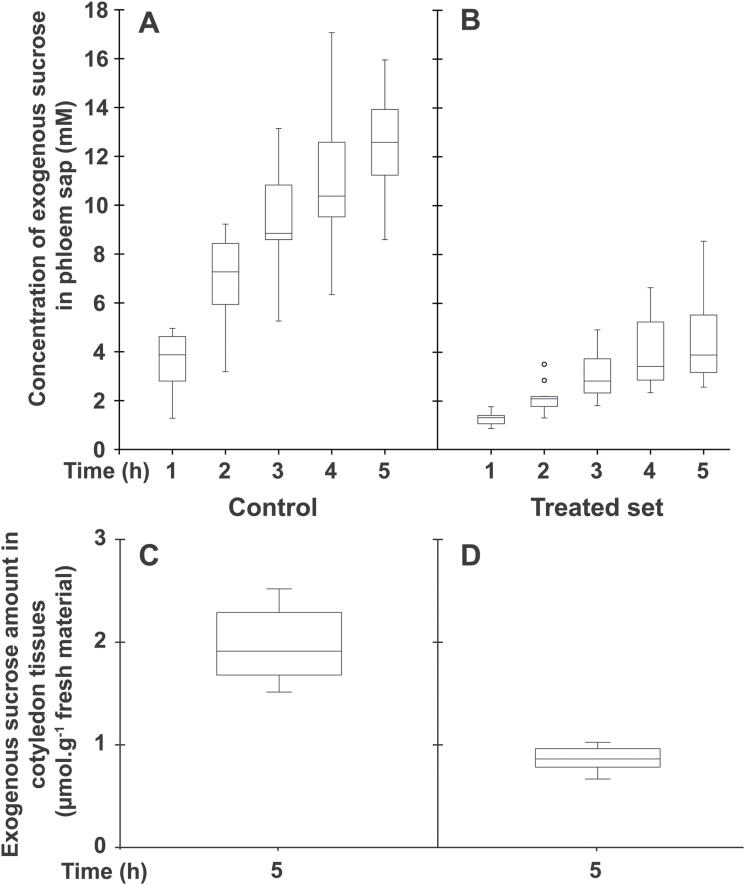
Effect of D-GFC on Suc uptake in the *Ricinus* model. Cotyledons were preincubated in a standard solution buffered at pH 5.0 for 30 min and then incubated in the same solution without (control; A, C) or with (treated set; B, D) 0.5 mM D-GFC. Thirty minutes later, [^14^C]Suc and unlabelled Suc were added to the solution to get 0.5 mM final concentration (specific activity: 0.04 mCi mmol^−1^; 10 ml per plant). After 30 min, the hypocotyl was severed at the hook region and the sap was collected every hour for 5 h and then analysed (A, B). At the end of experiment, the amount of exogenous Suc and metabolites (in Suc equivalent) in cotyledon tissues was determined by liquid scintillation counting (C, D). The Mann–Whitney test was used to assess statistically significant differences between the two sets at the 5% probability level: (A, B) except for *t*=1 h where *P*=0.0002, all *P*<0.0001; (C, D) *P*<0.0001. For box plots, *n*=10.

**Fig. 4.**
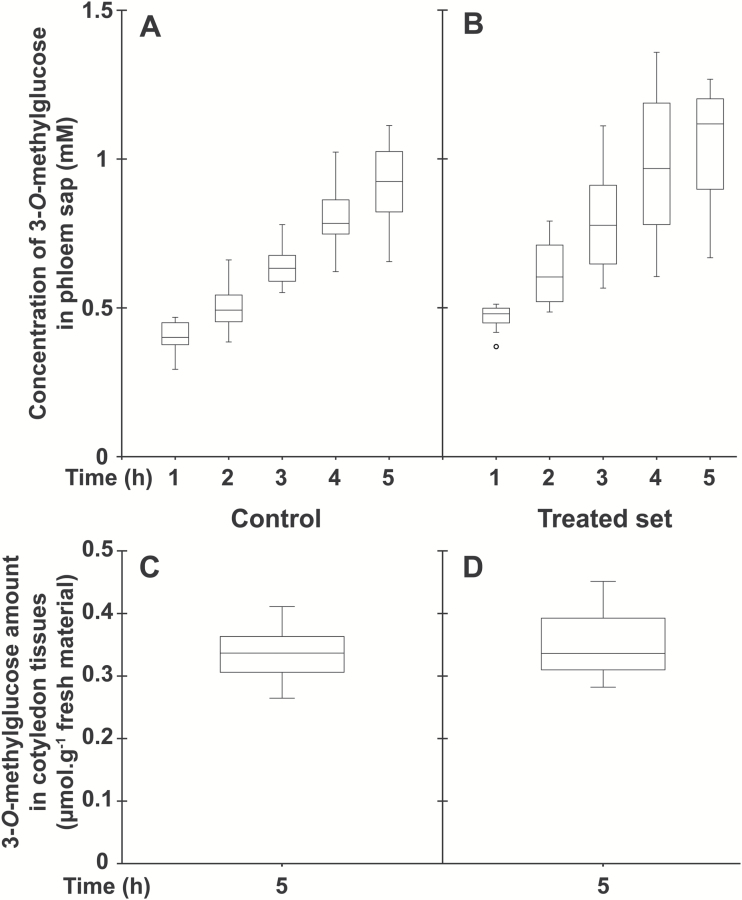
Effect of D-GFC on 3-*O*-MeG uptake in the *Ricinus* model. Cotyledons were preincubated in a standard solution buffered at pH 5.0 for 30 min and then incubated in the same solution without (control; A, C) or with (treated set; B, D) 0.5 mM D-GFC. Thirty minutes later, 3-*O*-[^3^H]MeG and unlabelled 3-*O*-MeG were added to the solution to get 0.5 mM final concentration (specific activity: 0.30 mCi mmol^−1^; 10 ml per plant). After 30 min, the hypocotyl was severed at the hook region and then the sap was collected every hour for 5 h and then analysed (A, B). At the end of experiment, the amount of 3-*O*-MeG (and metabolites) in cotyledon tissues was determined by liquid scintillation counting (C, D). The Mann–Whitney test was used to assess statistically significant differences between the two sets at the 5% probability level. (A, B) *t*=1h, *P*=0.009; *t*=2h, *P*=0.023; *t*=3h, *P*=0.052; *t*=4h, *P*=0.143; *t*=5h, *P*=0.075. (C, D) no statistically significant difference was noted. For box plots, *n*=10.

**Fig. 5.**
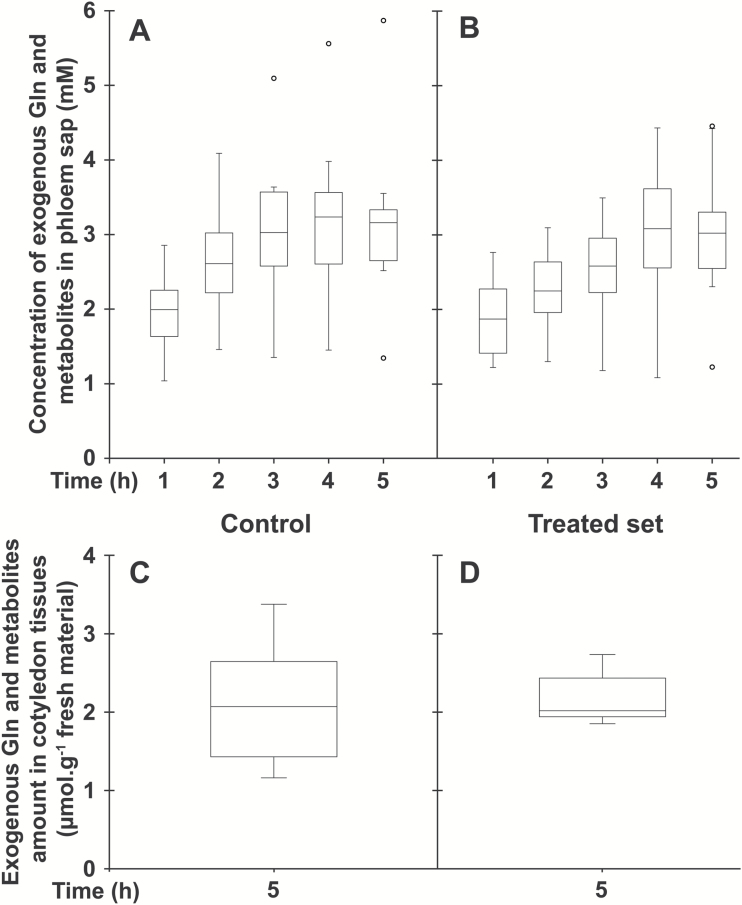
Effect of D-GFC on Gln uptake in the *Ricinus* model. Cotyledons were preincubated in a standard buffered solution at pH 5.0 for 30 min and then incubated in the same solution without (control; A, C) or with (treated set; B, D) 0.5 mM D-GFC. Thirty minutes later, [^3^H]Gln and unlabelled Gln were added to the solution to get 0.5 mM final concentration (specific activity: 0.30 mCi mmol^−1^; 10 ml per plant). After 30 min, the hypocotyl was severed at the hook region and then the sap was collected every hour for 5 h and then analysed (A, B). At the end of experiment, the amount of exogenous Gln and metabolites (in Gln equivalent) in cotyledon tissues was determined by liquid scintillation counting (C, D). The Mann–Whitney test was used to assess statistically significant differences between the two sets at the 5% probability level: for each time (A, B) and between the two sets (C, D), no statistically significant difference was noted. For box plots, *n*=10.

**Fig. 6.**
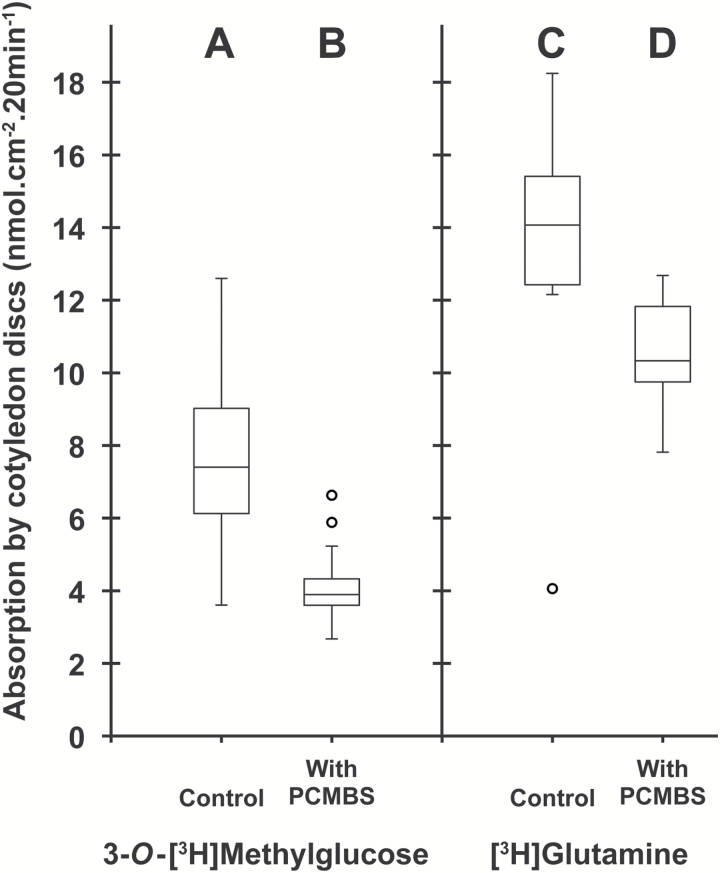
Effect of PCMBS on 3-*O*-MeG uptake or on Gln uptake by *Ricinus* cotyledon discs. Cotyledons were incubated in a standard buffered solution at pH 5.0 for 10 min, then in the same solution without (controls; A, C) or with (treated sets; B, D) 0.5 mM PCMBS for 10 min and then, for both sets, in the same solution containing 3-*O*-[^3^H]MeG and unlabelled 3-*O*-MeG (specific activity: 0.30 mCi mmol^−1^; 20 ml per 15 discs) (A, B) or [^3^H]Gln and unlabelled Gln (specific activity: 0.30 mCi mmol^−1^; 20 ml per 12 discs) (C, D) at a 0.5 mM concentration for 20 min. Under these short-time experimental conditions, PCMBS did not affect the transmembrane proton gradient in intact cotyledons (Rocher *et al.*, 2009). Furthermore, it was confirmed that 0.5 mM D-GFC exhibited no statistically significant effect on Gln uptake. The Mann–Whitney test was used to assess statistically significant differences between controls and treated sets at the 5% probability level. For 3-*O*-MeG, *P*=0.00014; for Gln, *P*=0.0014. For box plots, *n*=15 (3-*O*-MeG) or *n*=12 (Gln).

Considering that a relatively small amino acid conjugate (L-Lys–2,4D; *M*_r_=349.21) at 2.5 mM concentration was, in acidic conditions, an efficient inhibitor of uptake and phloem loading of various amino acids at a 1 mM concentration (Chollet *et al.*, 1997), we speculated that the large amphiphilic conjugate D-GFC could be used at low concentrations to affect the active uptake mediated by sugar carriers under their protonated form. Therefore, to compare the effects of D-GFC on uptake and phloem transport of the two sugars and the amino acid, the conjugate was used at 0.5 or 1 mM and exogenous Suc, 3-*O*-MeG, and Gln were used at 0.5 mM concentration in the incubation medium. Under these experimental conditions, D-GFC acted as a potent inhibitor of Suc uptake, especially Suc phloem transport. In a control set, exogenous Suc concentration in the phloem sap increased over time to reach a concentration factor (CF; concentration in the phloem sap/concentration in the incubation solution) of approximately 25 at the end of the experiment (Fig. 3A). In the treated set, the concentration of exogenous Suc in sieve tubes decreased by approximately 70% regardless of the time of sap collection (Fig. 3B). The amount of exogenous Suc and metabolites (in Suc equivalent) in cotyledon tissues collected at the end of the experiment (5 h) was reduced by 55% in the treated set (Fig. 3C, D). A slightly higher inhibition (65%; 0.96 and 0.34 µmol exogenous Suc g^−1^ fresh weight of *Ricinus* cotyledons in control and treated sets, respectively) occurred in a short-time experiment (1 h). As discussed below, Suc uptake by *Ricinus* cotyledons is also reduced by PCMBS (Orlich *et al.*, 1998). By contrast, D-GFC did not negatively affect the concentration of 3-*O*-MeG in the phloem sap, regardless of the time of phloem collection (Fig. 4A, B). This concentration increased over time to reach a CF of approximately 2 at the end of the experiment in both sets and was weakly but nevertheless significantly higher (Mann–Whitney test) in the treated set than in the control at times 1, 2, and 3 h. The amounts of 3-*O*-MeG and metabolites in cotyledon tissues were the same in both sets at 5 h (Fig. 4C, D). Similarly, the conjugate did not induce changes in the time course of accumulation of exogenous Gln and metabolites (in Gln equivalent) in the phloem sap. In both sets, their concentration plateaued during the last 2–3 h of the experiments to reach a CF of approximately 6 (Fig. 5A, B). In addition, the amounts of exogenous Gln and metabolites in cotyledon tissues were the same in control and treated sets (Fig. 5C, D). By contrast, 0.5 mM PCMBS reduced the uptake of 3-*O*-MeG in cotyledon tissues by 47% under short-time experiments (Fig. 6A, B). These experimental conditions were necessary because this thiol reagent progressively affects transmembrane proton gradients in *Ricinus* as mentioned below. At the same concentration, PCMBS moderately affected (approximately 25% inhibition) Gln uptake by tissues (Fig. 6C, D) but the inhibition was higher (≥50%) using plasma membrane vesicles from *Ricinus* cotyledons (Williams *et al.*, 1992).

**Fig. 7.**
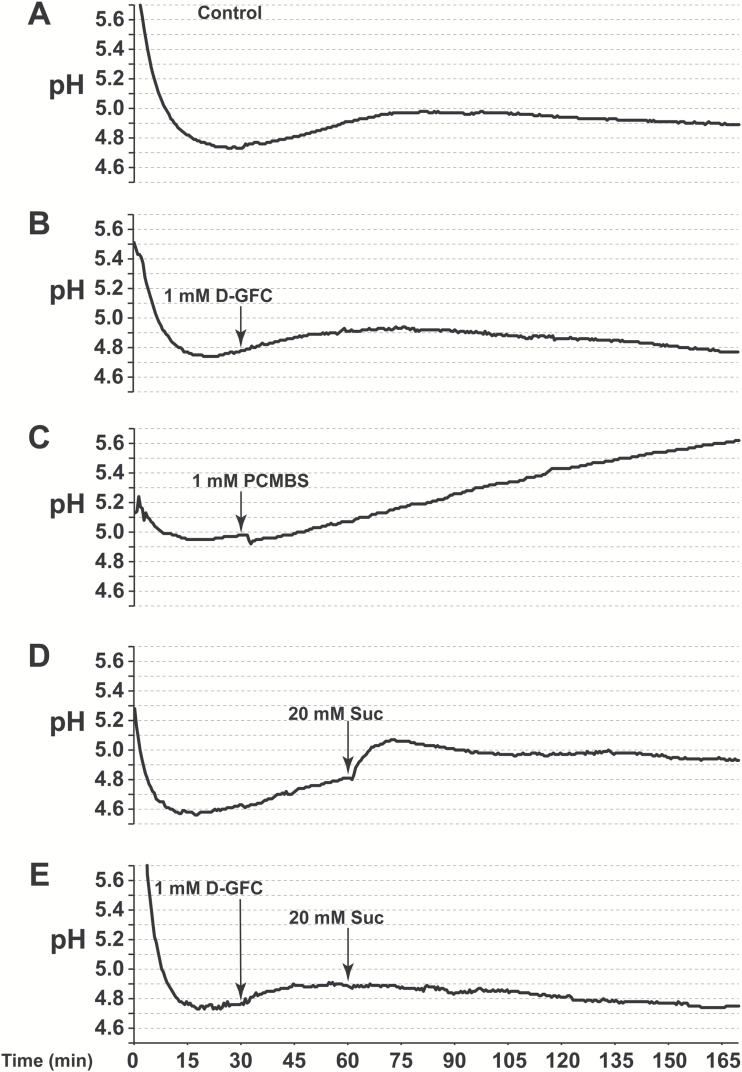
pH time course variation of the *Ricinus* incubation medium after different treatments. The cotyledons attached to the seedlings were incubated in a solution (10 ml) containing 0.25 mM MgCl_2_ and 0.5 mM CaCl_2_ at 27 ± 1 °C. The pH of the solution bathing the cotyledons decreased to values of 4.6–4.9 after 30 min and then stabilized between 4.8 and 5.0. Small aliquots of concentrated solutions of D-GFC, PCMBS or Suc at the same pH were added after reaching a steady state. (A) Control; (B) D-GFC (1 mM final concentration) was added at time 30 min; (C) PCMBS (1 mM final concentration) was added at time 30 min; (D) Suc (20 mM final concentration) was added at 60 min; (E) D-GFC (1 mM final concentration) was added at time 30 min then Suc (20 mM final concentration) was added at 60 min. Each experiment was repeated three times with similar results.

**Fig. 8.**
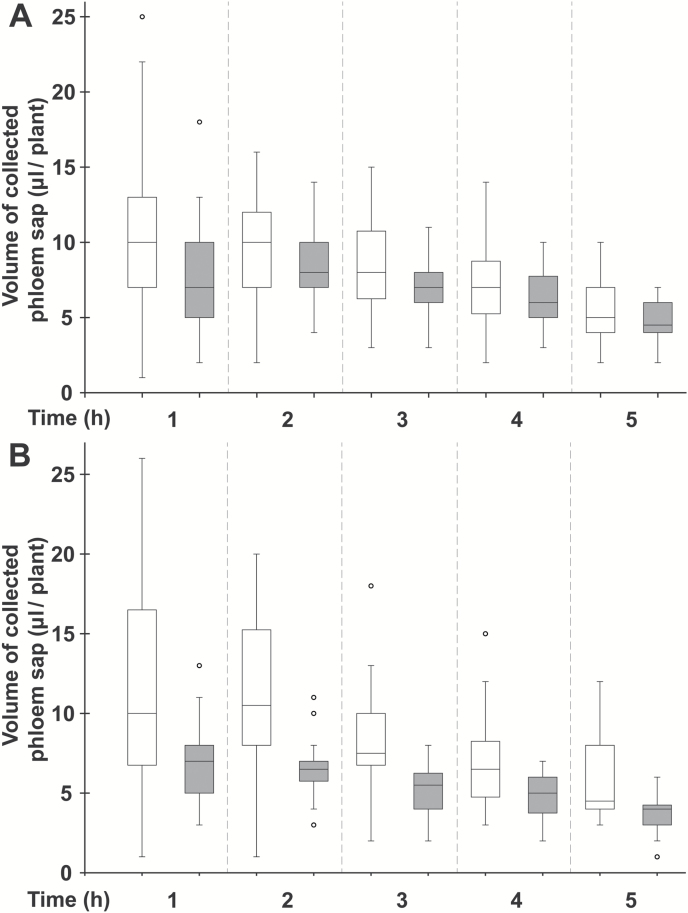
Effect of D-GFC on the volume of phloem sap collected. (A) Effect of 0.5 mM D-GFC on the volume of phloem sap collected during the experiments already described in Figs 3, 4 and 5. (B) Effect of 1 mM D-GFC on the volume of phloem sap collected. Controls: white boxes; treated sets: grey boxes. The Mann–Whitney test was used to assess statistically significant differences between the control and treated set at the 5% probability level: (A) Except for *t*=1 h where *P*=0.04, there were no significant differences between the control and treated set for all times; (B) *P*<0.05 for all times. For box plots, *n*=30 (A) or *n*=20 (B).

Considering these results, complementary experiments were performed to get additional data on D-GFC specificity and some insights on concentration and pH dependence, as well as post-treatment duration of Suc transport inhibition induced by the chemical. Although 0.5 mM D-GFC led to a dramatic inhibition of Suc phloem loading in *Ricinus*, the chemical was used at 1 mM in the following experiments. Under this experimental condition, D-GFC did not affect the time course of incubation medium acidification by cotyledon tissues (Fig. 7A, B) contrary to PCMBS (Fig. 7C). Therefore this result provided evidence that D-GFC did not alter the activity of the plasma membrane H^+^-ATPase via toxic effects on some cell functions, even for long-term experiments (several hours). In addition, 1 mM PCMBS quickly reduced the volume of phloem sap by 75% (Orlich *et al.*, 1998; Fig. 4B), while D-GFC had a less dramatic effect (from 32 to 42% inhibition) at the same concentration and a weak effect at 0.5 mM (Fig. 8A, B).

**Fig. 9.**
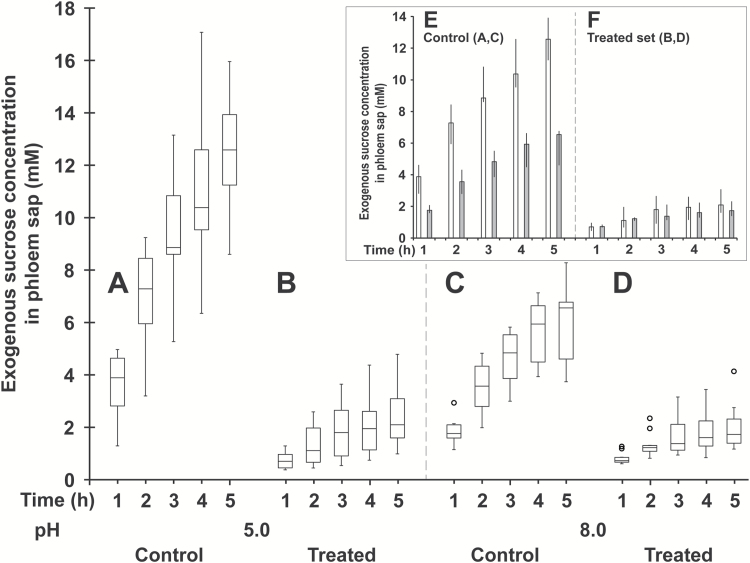
Time course of exogenous Suc and metabolites (in Suc equivalent) concentration in phloem sap of *Ricinus* in the absence (A, C) or presence (B, D) of 1 mM D-GFC. Cotyledons were preincubated in a standard solution buffered at pH 5.0 (A, B) or pH 8.0 (C, D) for 30 min and then incubated in the same solution containing 0 mM (A, C) or 1 mM (B, D) D-GFC. Thirty minutes later, [^14^C]Suc and unlabelled Suc were added to the solution to get 0.5 mM final concentration (specific activity: 0.04 mCi mmol^−1^; 10 ml per plant). After 30 min, the hypocotyl was severed at the hook region and then the sap was collected every hour for 5 h. At pH 5.0, the experiments with D-GFC at 0.5 (Fig. 3) and 1 mM concentration (this figure) were conducted together. Therefore, the control was the same for both figures. For a given pH and for each time point, the Mann–Whitney test was used to assess statistically significant differences between the control and the treated sets (A *vs* B and C *vs* D). In all cases, *P*<0.001. For box plots, *n*=10. Taking into consideration separately control sets (A, C) or treated sets (B, D), the same test was used to assess statistically significant differences for each time between pH 5.0 and pH 8.0 (see inset (E, F); median±interquartile range; pH 5.0 white columns, pH 8.0 grey columns). For control sets (E), *P*<0.005 for all times; for treated sets (F), there were no statistically significant differences between pH 5.0 and 8.0 for all times.

**Fig. 10.**
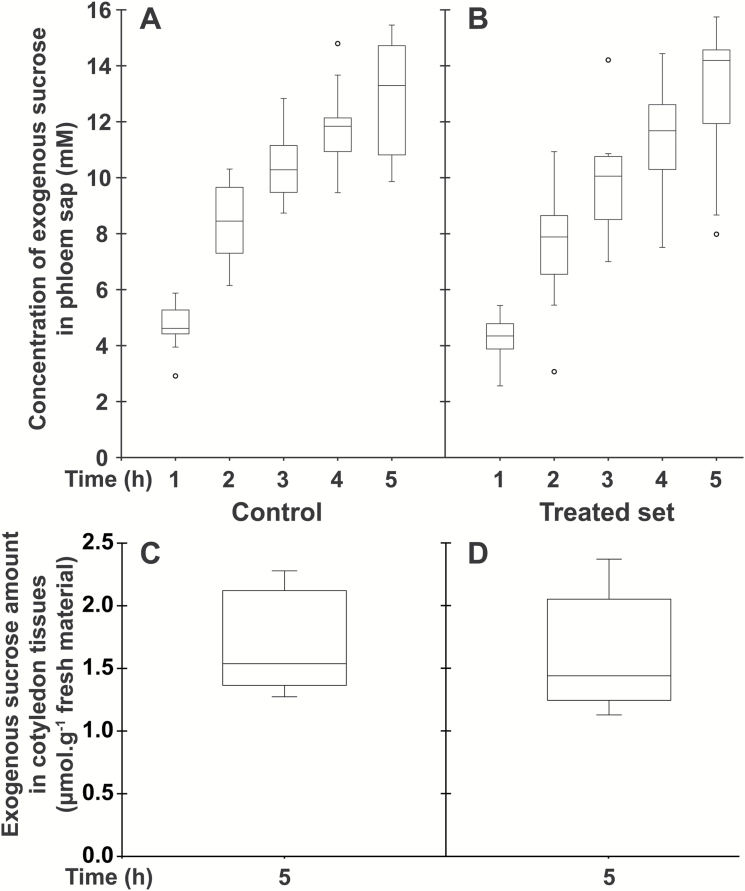
Reversibility of the inhibitory effect of D-GFC on Suc uptake in the *Ricinus* model. Cotyledons were incubated in a standard buffered solution at pH 5.0 for 30 min and then in the same solution without (control; A, C) or with (treated set; B, D) 0.5 mM D-GFC for 60 min. Cotyledons were then washed 3 × 2 min with the standard buffer solution and were incubated (control and treated set) in the standard solution containing 0.5 mM [^14^C]Suc and unlabelled Suc (final concentration, specific activity: 0.04 mCi mmol^−1^; 10 ml per plant). After 30 min, the hypocotyl was severed at the hook region and the sap was collected every hour for 5 h and then analysed (A, B). At the end of experiment, the amount of [^14^C]Suc (and labelled metabolites) in cotyledon tissues was determined by liquid scintillation counting (C, D). The Mann–Whitney test was used to assess statistically significant differences between the two sets at the 5% probability level. (A, B) For each time, no significant differences were noted; (C, D) no significant difference was noted. For box plots, *n*=10.

Increasing conjugate concentration from 0.5 to 1 mM in the incubation medium buffered at pH 5.0 led to an accentuated decrease of exogenous Suc concentration in the phloem sap (80–85% *vs* 70%) regardless of the time of sap collection (compare Fig. 3A, B and Fig. 9A, B). The same was observed for the amounts of exogenous Suc and metabolites in cotyledon tissues collected at the end of experiments (approximately 70% *vs* 55%) (compare Fig. 3C, D and Table 1). The uptake and phloem transport of exogenous Suc were either markedly dependent (control sets) or independent (treated sets) of the incubation medium pH. In control sets, the concentration of exogenous Suc in the sap and its amount in cotyledon tissues at 5 h were both reduced by approximately 50% from pH 5.0 to 8.0 (Fig. 9A, C, E; Table 1). By contrast, in the treated set, the low exogenous Suc contents in these two compartments were unaffected (Fig. 9B, D, F) or were weakly affected (Table 1) by the pH change, suggesting that 1 mM D-GFC reduced to nothing, or almost nothing, the pH-dependent component of the active Suc transport, i.e. the symport H^+^–Suc. This well-known mechanism described several decades ago (Komor, 1977; Komor *et al.*, 1977; Hutchings, 1978; Malek and Baker, 1978; Delrot and Bonnemain, 1979, 1981) was notably supported by an alkalinization of the incubation medium due to concomitant influxes of protons and Suc in cotyledon tissues. At 1 mM, D-GFC completely abolished the transient alkalinization induced by these influxes after the addition of 20 mM Suc (final concentration) in the incubation solution (Fig. 7D, E). The inhibition of the active component of Suc transport was thoroughly removed after a short washing (3 × 2 min) of cotyledon tissues in the standard medium. Besides previous incubation of tissues for 1 h in a solution containing 1 mM conjugate (treated set) before washing, the time course enrichment of exogenous Suc in the phloem sap (Fig. 10A, B) and Suc uptake by cotyledon tissues (Fig. 10C, D) were similar in the control and treated sets.

**Table 1.** *Effect of 1 mM D-GFC on 0.5 mM Suc uptake (µmol g* ^*−1*^ *fresh material) by* Ricinus *cotyledon tissues at pH values of 5.0 and 8.0* Cotyledons were preincubated in a standard solution buffered at pH 5.0 or pH 8.0 for 30 min and then incubated in the same solution containing 0 mM (Control) or 1 mM (Treated set) D-GFC (10 ml per plant). Thirty minutes later, [^14^C]Suc and unlabelled Suc were added to the solution to get 0.5 mM final concentration (specific activity: 0.04 mCi mmol^−1^). After 30 min, the hypocotyl was severed at the hook region and then the sap was collected every hour for 5 h (see Fig. 9). At the end of experiment, the amount of exogenous Suc and metabolites (in Suc equivalent) in cotyledon tissues was determined by liquid scintillation counting. The data are means±95% CI, *n*=10.

pH 5.0			pH 8.0		
Control	Treated set	Inhibition (%)	Control	Treated set	Inhibition (%)
1.98 ± 0.23	0.59 ± 0.08	−70	0.97 ± 0.10	0.79 ± 0.05	−19

While PCMBS forms covalent bonds with protein cysteine residues, D-GFC acts as a reversible inhibitor like the natural glucoside phlorizin (Bush, 1993). The thiol reagent and D-GFC exhibit similarities and differences. The most astonishing similarity is that the conjugate (described here) and PCMBS (Orlich *et al.*, 1998) inhibit Suc transport in *Ricinus* by 80–90% at the same concentration (1 mM). The most striking difference is the specificity of D-GFC, which does not affect Gln or 3-*O*-MeG transport in cotyledon tissues, contrary to PCMBS. In addition, the xenobiotic glucoside does not change the time course acidification of the incubation medium by cotyledon tissues while the sulfhydryl reagent alters this process (Williams and Hall, 1987; Williams *et al.*, 1990; our data). It has been well known for decades, first by using plant tissues and then plasma membrane vesicles or complemented yeast mutants, that PCMBS inhibits not only Suc carriers (Delrot *et al.*, 1980; Bush, 1993; Lemoine, 2000; Barth *et al.*, 2003; Sauer, 2007) and oligopeptide carriers (Jamai *et al.*, 1994; Rentsch *et al.*, 1995), but also amino acid transporter systems to various degrees (from 5 to 75%) (Servaites *et al.*, 1979; Despeghel and Delrot, 1983; M’Batchi and Delrot, 1984; Montamat *et al.*, 1999). In that respect, some data emerged from *Ricinus* studies on the effect of PCMBS on nutrient uptake and the activity of the PM H^+^-ATPase, especially during the 1980s and 1990s (Lorenc-Plucinska and Ziegler, 1987; Williams *et al.*, 1992, 1996; Weig and Komor, 1996; Marvier *et al.*, 1998). The effect of PCMBS on hexose transport is variable (from 0 to 70% inhibition) according to the plant material studied (Maynard and Lucas, 1982; Felker and Goodwin, 1988; Renault *et al.*, 1992). It can also react with many PM intrinsic proteins involved in the phloem transport of acidic organic compounds, such as salicylic acid (Rocher *et al.*, 2009) and auxin (Tamas and Davies, 2016), as well as mineral nutrient uptake, notably K^+^ (Wilkinson and Ohki, 1991; Smart *et al.*, 1996). Therefore, it is not surprising that PCMBS reduces phloem sap exudation in *Ricinus* much more (Orlich *et al.*, 1998) than D-GFC (Fig. 8).

### Effect of D-glucose–fenpiclonil conjugate on phloem loading of endogenous sugars in *Ricinus* seedlings

**Fig. 11.**
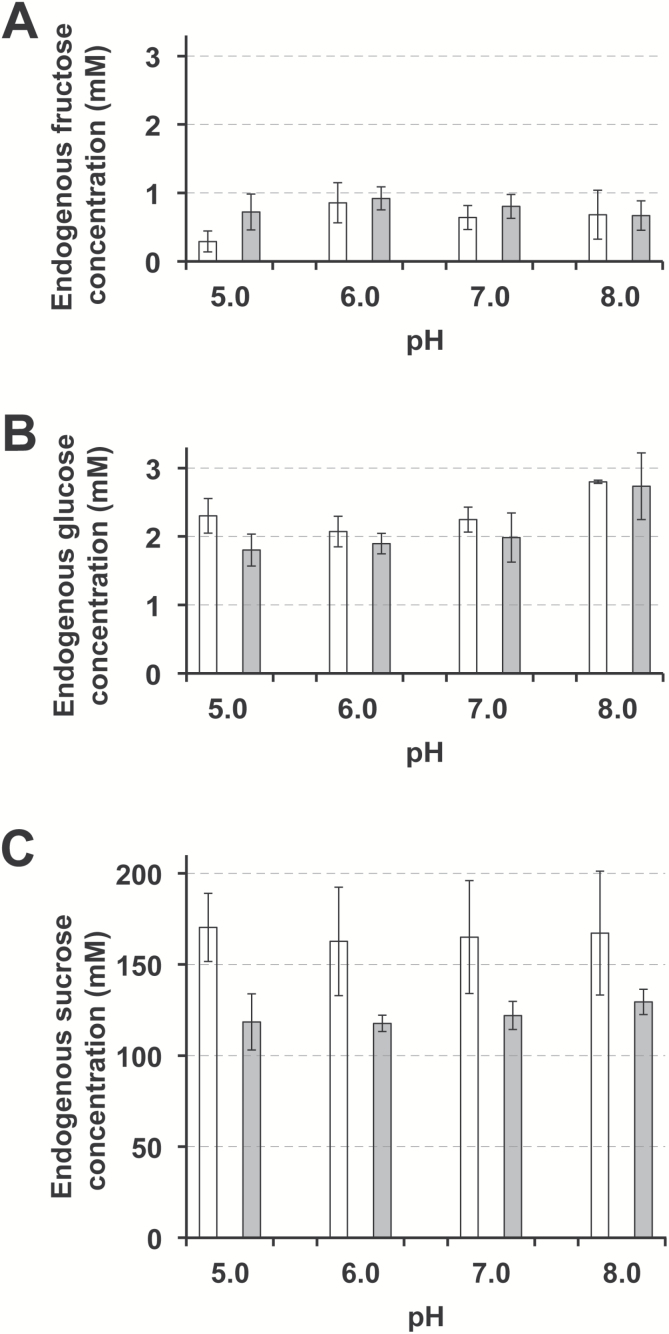
Fru (A), Glc (B), and Suc (C) concentrations in the phloem sap of *Ricinus* at different pH values and in the absence (white columns) or presence (grey columns) of D-GFC. Cotyledons were preincubated in a standard buffered solution at pH 5.0, 6.0, 7.0, or 8.0 for 30 min and then incubated in the same solution containing 0.5 mM D-GFC. After 30 min, the hypocotyl was severed at the hook region and then the sap was collected during the fifth hour after the removal of endosperm from cotyledons and then analysed. Mean of three sets of seven plants each ±95% CI.

Because of its high specificity, at least in the heterotrophic tissues studied (Figs 3, 4, 5 and 7), D-GFC should be a suitable tool for long-term studies (at least several hours) on endogenous sugar transport and compartmentation. Therefore, we used it to investigate the pattern of sugar phloem loading during the fifth hour after the removal of endosperm from cotyledons, i.e. when Suc in the phloem sap derives uniquely from starch breakdown (Kallarackal *et al.*, 1989; Orlich *et al.*, 1998). In control sets, the concentrations of fructose (which exhibited large variations due to detection limits) and glucose in the sap (Fig. 11A, B) were similar to those previously reported (Kallarackal and Komor, 1989). Glucose amounts did not change markedly in response to the different pH values of the incubation medium (from pH 5.0 to 8.0). Similarly, the Suc concentration (whose values oscillated around 160 mM from the third to the sixth hour after endosperm removal in our plant material) remained stable regardless of the pH values of the incubation medium (Fig. 11C). The conjugate at 0.5 mM did not affect total hexose concentration from pH 5.0 to pH 8.0. In contrast, it reduced the concentration of Suc in the phloem sap from 30.4% (pH 5.0) to 22.6% (pH 8.0) (Fig. 11C). The data can be analysed in relation to the pattern of solute phloem loading in *Ricinus*.

Three different pathways have been considered for Suc transport from the endosperm (or incubation solution) to the companion cell–sieve element complex: a direct apoplasmic route, a symplasmic route and an indirect apoplasmic route (Orlich and Komor, 1992; Orlich *et al.*, 1998). This phloem loading pattern has been supported by structural, physiological, and molecular data. On the one hand, the high expression of the plasma membrane H^+^-ATPase (Williams and Gregory, 2004) and a Suc carrier (Bick *et al.*, 1998) in the phloem, and the relative paucity of plasmodesmata between mesophyll and bundle sheath (Orlich *et al.*, 1998) are in agreement with a direct apoplasmic component of phloem loading of Suc exported from the endosperm. On the other hand, the lower epidermis cells are modified into transfer cells (Bick *et al.*, 1998) that possess (i) the proton pumping machinery and the Suc carrier equipment necessary for efficient sugar uptake from the endosperm (Bick *et al.*, 1998; Williams and Gregory, 2004) and (ii) high symplasmic connections with the mesophyll allowing cell to cell transport via plasmodesmata, at least to the proximity of the bundle sheath. In addition, a transient phloem loading of Suc occurs in the presence of PCMBS (Orlich *et al.*, 1998). These data support the involvement of a symplasmic component. Finally, time course analyses of labelled and non-labelled Suc in three compartments (mesophyll symplasm, cell-wall space and phloem exudate) indicate that an indirect apoplastic phloem loading occurs (Orlich and Komor, 1992).

Under our experimental conditions, the direct apoplasmic phloem loading and the primary symplasmic route from epidermal cells cannot operate because of the removal of the endosperm for several hours (Orlich and Komor, 1992). The involvement of a supplementary symplasmic route from storage compartments is supported by the lack of effect that pH values (from 5.0 to 8.0) of the incubation medium have on the endogenous Suc concentration in the phloem sap. In addition, a contribution of the indirect apoplasmic route is demonstrated by the slightly pH-dependent inhibition of endogenous Suc loading by the conjugate (Fig. 11C). Considering that the inhibition of Suc phloem loading by D-GFC is not optimal at 0.5 mM (compare Figs 3B and 9B), the contributions of the indirect apoplasmic route and the symplasmic route should constitute approximately one-third and two-thirds of phloem loading, respectively, under our experimental conditions (pH 5.0, fifth hour after the removal of endosperm). Therefore our data (i) support previous work (Orlich and Komor, 1992; Orlich *et al.*, 1998) and (ii) allow a quantitative approach to the relative contribution of these two latter ways.

### Effect of D-glucose–fenpiclonil conjugate on Suc transport in two other biological models

Experiments were conducted on *Vicia faba* leaf tissues and *Saccharomyces cerevisiae* cells transformed with *AtSUC2*, which encodes the sucrose transporter involved in sucrose phloem loading in Arabidopsis (Stadler and Sauer, 1996). *Vicia faba* is typically an apoplasmic phloem loader. The maturation of leaves from importing to exporting stages is characterized by a marked reduction of symplasmic connexions between the phloem and the mesophyll and, within the phloem itself, by an additional symplasmic isolation of the companion cell–sieve element complex (Bourquin *et al.*, 1990). Furthermore, in mature leaves, the companion cells are modified into transfer cells exhibiting a high expression and polarized addressing of the PM H^+^-ATPase (Bouché-Pillon *et al.*, 1994). The effects of the conjugate on Suc and 3-*O*-MeG uptake by leaf discs were compared with data previously published concerning PCMBS (M’Batchi and Delrot, 1984) and phlorizin (Lemoine and Delrot, 1987) (Table 2). The dramatic inhibition of Suc uptake induced by 0.5 mM D-GFC in *Ricinus* was observed again using Fabaceae leaf discs. By comparison, 5 mM phlorizin is a relatively poor inhibitor. The effect of D-GFC in Fabaceae was not as specific as in *Ricinus* because of its slight effect on 3-*O*-MeG uptake. Nevertheless, considering the Suc/3-*O*-MeG inhibition ratio, its specificity is better than that of PCMBS and clearly of phlorizin (Table 2).

**Table 2.** Effect of D-GFC, PCMBS and phlorizin on the uptake of Suc and 3-O-MeG by broad bean leaf discs In the present study, leaf discs were preincubated in a standard buffer solution (pH 5.0) for 30 min. After preincubation, the discs were transferred to the same medium solution without (control) or with 0.5 mM D-GFC or 0.5 mM PCMBS in the presence of 0.5 mM labelled Suc (specific activity: 0.20 mCi mmol^−1^; 20 ml per 15 discs) or 3-*O*-MeG (specific activity: 0.30 mCi mmol^−1^; 20 ml per 12 discs) for 30 min. Radioactivity measurements were made on each disc separately. The medians were used to calculate the inhibition percentages relative to control discs.

Inhibitor	Substrate	Inhibition (%)	Reference
0.5 mM D-GFC (pH 5.0)	0.5 mM Suc	85.0	Present study
	0.5 mM 3-*O*-MeG	17.2	Present study
0.5 mM PCMBS (pH 5.0)	0.5 mM Suc	71.4	Present study
	0.5 mM 3-*O*-MeG	19.1	Present study
0.5 mM PCMBS (pH 6.0)	1 mM Suc	73.7	M’Batchi and Delrot (1984)
	1 mM 3-*O*-MeG	23.6	M’Batchi and Delrot (1984)
5 mM phlorizin (pH 5.0)	1 mM Suc	53	Lemoine and Delrot (1987)
	1 mM 3-*O*-MeG	23	Lemoine and Delrot (1987)

Transgenic *Saccharomyces cerevisiae* cells transformed with *AtSUC2* constituted an elegant model to study the effect of D-GFC on Suc uptake by the H^+^–Suc symporter involved in Suc loading in Arabidopsis (Stadler and Sauer, 1996; Gottwald *et al.*, 2000). At a concentration as low as 0.25 mM, the active component of the Suc uptake was inhibited by approximately 50% in 5 min. The maximum inhibition plateaued (approximately 80%) at 0.5 mM (Table 3).

**Table 3.** *Effect of D-GFC on the uptake of Suc into transgenic* Saccharomyces cerevisiae *cells* Uptake of Suc into *Saccharomyces cerevisiae* cells transformed with *AtSUC2* or an empty vector in the presence of 0 mM (control), 0.25 mM, 0.5 mM, and 1mM D-GFC for 5 min. The [^14^C]Suc concentration was 0.5 mM in all experiments at pH 4.5 (specific activity: 0.50 mCi mmol^−1^). Data were expressed as the mean±95% CI (*n*=4). The experiment was repeated twice with similar results using 0.5 mM D-GFC

D-GFC concentration	Suc uptake (nmol min^−1^ mg cells^−1^)			Inibition^*b*^ (%)
	Empty vector	*AtSUC2*	Active uptake^*a*^	
Control (0 mM)	0.19 ± 0.08	0.91 ± 0.03	0.71	—
0.25 mM	0.17 ± 0.03	0.55 ± 0.05	0.37	47.9%
0.5 mM	0.21 ± 0.09	0.34 ± 0.04	0.13	81.5%
1 mM	0.13 ± 0.02	0.28 ± 0.01	0.15	79.1%

^*a*^ The active uptake of Suc was calculated from the difference between the two *S. cerevisiae* cells (empty vector and *AtSUC2*).

^*b*^ The D-GFC-induced inhibition was expressed as the percentage of active uptake/control uptake.

These data indicate that the potent inhibitory effect of D-GFC on sucrose carriers is not limited to cotyledons of seedlings with endosperm, i.e. heterotrophic tissues that function as the small intestinal wall (Robinson and Beevers, 1981*a*). The effect of D-GFC is similar to that of PCMBS in apoplasmic loaders and much higher than the natural glucoside phlorizin (Lemoine and Delrot, 1987; Bush, 1993).

## Conclusion

Using different biological models (heterotrophic cotyledon tissues, mature exporting leaves, and transgenic *Saccharomyces cerevisiae* cells) from different plant families (Euphorbiaceae, Fabaceae, and *AtSUC2* from Brassicaceae), our data show that D-GFC is an inhibitor of Suc carriers as potent as PCMBS in acidic conditions. This xenobiotic glucoside reversibly blocks the H^+^–Suc symporters involved in Suc exchanges at strategic sites of the plant, namely at the triploid endosperm–cotyledon tissues interface and phloem loading in cotyledons, as well as phloem loading in mature leaves of apoplasmic loaders. While PCMBS forms covalent bonds with sulfhydryl groups of many PM intrinsic proteins and therefore affects uptake and phloem transport of many solutes in addition to Suc, D-GFC can exhibit much more selectivity, especially in *Ricinus* seedlings and possibly in other seedlings with endosperm. Unlike PCMBS, this new tool in phloem biology allows long-term phloem exudation and therefore investigation, with quantitative analysis, of the pathways involved in phloem loading of endogenous Suc in *Ricinus*, as evidenced in the present work. The use of D-GFC can be extended to the study of sugar exchange between vascular tissue apoplasm and symplasm in response to abiotic stresses.

## Abbreviations:

CF: concentration factor
Gln: glutamine
D-GFC: D-glucose–fenpiclonil conjugate
3-*O*-MeG: 3-*O*-methylglucose
PCMBS: *p*-chloromercuribenzenesulfonic acid sodium salt
PM: plasma membrane
Suc: sucrose.
